# Temporal Variation in the Abundance and Richness of Foliage-Dwelling Ants Mediated by Extrafloral Nectar

**DOI:** 10.1371/journal.pone.0158283

**Published:** 2016-07-20

**Authors:** Ceres Belchior, Sebastián F. Sendoya, Kleber Del-Claro

**Affiliations:** 1 Programa de Pós-Graduação em Ecologia e Conservação de Recursos Naturais, Instituto de Biologia, Universidade Federal de Uberlândia, Uberlândia MG, Brazil; 2 Departamento de Biologia Animal, Instituto de Biologia, Universidade Estadual de Campinas, C.P. 6109, Campinas SP, Brazil; Universidade de São Paulo, Faculdade de Filosofia Ciências e Letras de Ribeirão Preto, BRAZIL

## Abstract

Plants bearing extrafloral nectaries (EFNs) are common in the Brazilian cerrado savanna, where climatic conditions having marked seasonality influence arboreal ant fauna organization. These ant-plant interactions have rarely been studied at community level. Here, we tested whether: 1) EFN-bearing plants are more visited by ants than EFN-lacking plants; 2) ant visitation is higher in the rainy season than in dry season; 3) plants producing young leaves are more visited than those lacking young leaves in the rainy season; 4) during the dry season, plants with old leaves and flowers are more visited than plants with young leaves and bare of leaves or flowers; 5) the composition of visiting ant fauna differs between plants with and without EFNs. Field work was done in a cerrado reserve near Uberlândia, MG State, Brazil, along ten transects (total area 3,000 m^2^), in the rainy (October-January) and dry seasons (April-July) of 2010–2011. Plants (72 species; 762 individuals) were checked three times per season for ant presence. Results showed that 21 species (29%) and 266 individuals (35%) possessed EFNs. These plants attracted 38 ant species (36 in rainy, 26 in dry season). In the rainy season, plants with EFNs had higher ant abundance/richness than plants without EFNs, but in the dry season, EFN presence did not influence ant visitation. Plant phenology affected ant richness and abundance in different ways: plants with young leaves possessed higher ant richness in the rainy season, but in the dry season ant abundance was higher on plants possessing old leaves or flowers. The species composition of plant-associated ant communities, however, did not differ between plants with and without EFNs in either season. These findings suggest that the effect of EFN presence on a community of plant-visiting ants is context dependent, being conditioned to seasonal variation.

## Introduction

Interspecific interactions are considered important processes that can influence species variation and adaptation [1] as well as community organization and stability [2–4]. Systems consisting of interactions between plants and insects possess much variation in the identity of interacting species, representing good models for study of temporal and spatial differences (see examples in [5]). Many studies addressing abundance, diversity and behavior of ants on the vegetation are available [6–8]. There is strong evidence to consider ant-plant interactions as ‘keystone interactions’ in many communities, especially in the tropics [5, 9–12].

The presence of renewable and predictable food sources on the vegetation favors its use as substrate for foraging by ants and explains the high frequency of interactions observed in these systems [5, 9, 13]. Among the available options, ants can use extrafloral nectaries (EFNs), which are glands that secrete nectar rich in sugar, water and amino acids. These glands are very diverse in morphology, possessing structures that can differ considerably from floral nectaries in size and shape [14–15]. In Brazil, records of EFNs on the flora of many biomes are frequent [5, 8, 16], especially in the cerrado savanna, where the frequency and abundance of plants with EFNs are relatively high [5, 6, 17]. For instance, a total of 45 EFN-bearing woody species from 17 families were sampled in 10 areas of cerrado in southeast and west Brazil, and quantitative samplings of the local floras revealed that 15 to 25% of the species were found to possess EFNs, accounting for up to 31% of the individuals surveyed [8, 17]. Generally, ants are the main visitors of EFNs, among other arthropods, like wasps, flies, spiders, bees, beetles and mites [18–20].

The availability of highly energetic liquid resources on the vegetation, such as extrafloral nectar, can influence ant survival [21] and community organization, and can promote higher richness/abundance of ants on plants with EFNs compared to those without [9]. However, the mechanisms that explain patterns of arboreal ant fauna in the cerrado savanna have yet to be elucidated [8] and the effects of EFN-plant community on ant community need further investigation [7].

Studies in Neotropical regions have suggested that changes in associations between ants and plants with EFNs can be related to the existence of climatic conditions having striking seasonality [5, 22]. Thus, seasonal variation could also affect the way community is structured over time. In the Brazilian cerrado, variations in the abundance of insects throughout the seasons are common [23–25]. Seasonality can influence the availability of food resources to the ants [26–28], and can also make the EFNs more important during certain periods [8, 29, 30]. Additionally, the phenological conditions of plants can influence insect-plant interactions if the offering of resources, especially extrafloral nectar, varies with phenological changes in plants [31, 32].

To understand the role and adaptive significance of EFN-mediated ant-plant associations in a community, it is necessary to investigate the distribution of plants with EFNs in the flora and its visiting ant fauna [22]. In the cerrado savanna, many manipulative studies investigated the importance of EFNs as a biotic defense mechanism focusing on a particular plant species and its associated ant species [5]. On the other hand, comparative analyses of interactions between ants and plants addressing the influence of seasonality and presence of EFNs in the characteristics of community are scarce [8, 30, 32].

In the cerrado vegetation, new leaves are very abundant and possess more active EFNs during the rainy season, while in the dry season leaves with active EFNs are not common, as well as the volume of extrafloral nectar produced is reduced [30–33]. A recent study of Camarota and co-workers [34] addressed whether competition for extrafloral nectar exerts a significant influence on the structure of ant community on cerrado vegetation, and whether this influence changes across the contexts of extrafloral nectar availability. They predicted that some ant species would prevail under contexts where nectar availability is greater, with consequences for overall patterns of species richness and composition.

In the present study, we evaluated whether EFN-plants are important to structure the visiting ant community on the local flora and how these interactions vary over time. The main objectives were to verify whether there are seasonal differences in terms of richness and abundance of visiting ants, whether phenological factors affect the presence of these visitors and to compare the composition of ant fauna between plants with and without EFNs. The following hypotheses were tested: 1) EFN-bearing plants are more visited than EFN-lacking plants, because sugar sources are vital to ant nutrition and colony survivorship; 2) Ant visitation on plants with EFNs is higher during the rainy season, because there are more plants offering extrafloral nectar during this period than in the dry season; 3) EFN-bearing plants with young leaves are more visited than EFN-lacking plants with young leaves during the rainy season, because extrafloral nectar is a valuable resource to ants, and plants could be more vulnerable to herbivores during this period; 4) in the dry season, plants with remaining leaves or flowers are more visited than plants with young leaves or bare of leaves and flowers, as a shortage of extrafloral nectar increases the importance of other resources to ants; 5) the composition of visiting ant fauna differs between plants with and without EFNs, because ant species that depend on extrafloral nectar would tend to visit plants containing this resource more frequently.

## Materials and Methods

### Study site

Field work was conducted between August 2010 and July 2011 (90 days and 600 hours of sampling efforts) in a private natural savanna reserve (Clube Caça e Pesca Itororó de Uberlândia/CCPIU—48°17’ W; 18°58’ S) in Uberlândia, Minas Gerais State, southeastern Brazil. The Biology Institute of Universidade Federal de Uberlândia has a memorandum of understanding with CCPIU, an agreement between Mr. Nilson Dias, head of CCPIU, and Dr. Kleber Del-Claro, director of Biology Institute, that enables ecological studies in the area. The vegetation is dominated by cerrado *strictu sensu*, consisting of a woody layer of trees and large shrubs 2–8 m tall, and a ground layer composed of grasses, herbs and small shrubs. Köppen [35] classifies the Uberlândia climate as tropical altitude with hot summer (Cwa), characterized by two well-defined seasons: rainy summer (October to March) and dry winter (April to September).

### Sampling design and data collection

In a cerrado *strictu sensu* area, shrubs and trees 1–5 m tall were selected randomly along five transects of 50 x 4 m and five transects of 100 x 4 m, apart from each other by at least 50 m. For this selection, transects were divided into plots of 1 m^2^, resulting in 200 or 400 plots, depending on transect size. The plots on each transect were numbered, and 50 or 100 plots per transect, totaling 750, were chosen by drawing pieces of numbered paper. In the field, one plant was marked in each plot, but there were cases in which two plants were marked because stems were very close to each other. This procedure added 12 plants, totaling 762 studied plants.

To record abundance and richness of visiting ants, each plant was sampled once in the morning (7h30–10h30), once in the afternoon (13h30–16h30) and once at night (traps were kept over the course of 14 hours from 17h00 until 7h00), to ensure a better representation of ant fauna. Sampling was carried out once per season for each plant. Samplings in the morning, afternoon and night for plants of the same transect were carried out within a maximum of three days. This procedure lasted 45 days and 300 hours per season and was designed for detecting differentiation in ant visitation between the seasons. Samplings were made avoiding cloudy days or days with precipitation both in rainy (October 4, 2010 –January 27, 2011) and dry seasons (April 25 –July 26, 2011). Air temperature across days varied between 25–35°C in rainy season and between 20–30°C in dry season. Additionally, phenological state of the plants was registered in both seasons by recording whether plants had old leaves, young leaves, flowers (including floral buds) and/or fruits [30].

Ant sampling during the morning and afternoon periods was conducted by direct observation, and each plant was observed for five minutes. All ants observed on the plants, in any position 30 cm above ground, were registered through instantaneous counting. To avoid double counting of co-specific individuals, plant’s parts were observed separately and ants were counted as they were seen (‘snapshot’ [36]). Ant sampling during the night period was performed using adhesive traps, made by transparent plastic sheets covered with honey cold wax (Depil Bella^®^), that were fixed with plated pins (size #29) around the stem at 30 cm above the ground—the adhesive traps stayed on the plants for 14 hours. Plants possessing girth up to 10 cm, measured at 30 cm the ground, received traps of 10 x 7 cm, while plants possessing girth between 10 and 20 cm received traps of 20 x 7 cm. For plants possessing girth greater than 20 cm, the two kinds of trap were used together.

At least one individual from each ant species observed by plant was collected and placed in 70% ethanol. Identification of ant species was carried out by comparing the collected individuals with those specimens identified at the Museu de Zoologia da Universidade de São Paulo. Voucher specimens were deposited at the Museu da Biodiversidade do Cerrado at the Universidade Federal de Uberlândia. Identification of plant species was mostly carried out in the field as most plants were in a vegetative state. Voucher specimens were deposited at the Herbarium Uberlandense at Universidade Federal de Uberlândia.

### Data analyses

To determine whether ant visitation levels varied between plants with or without EFNs, between seasons and according to plant phenological factors, we constructed generalized linear mixed models (GLMMs). Sampling transect and plant species were factors grouping and reducing independence of our sampling units (individual plants), and hence were included as random variables. Because we aimed to estimate total ant activity on the plant (and not to focus on any particular period), ant visitation levels at the scale of individual plants were measured as the total number of individuals and ant species observed per plant over a full day. Although differences in sampling methods between day and night may affect absolute values of each period, this effect is shared for all categories of our predictive variables. Separated models were constructed for ant abundance and richness per plant, which were treated as response variables. In these models, the effects of sampling season and presence of EFNs were used as fixed variables. In both models we also included the phenological factors (old leaves, young leaves, flowers and fruits) as fixed variables modeling either ant abundance or richness. Given that the response variable was a count, and to avoid overdispersion, we fitted models with a negative binomial distribution correcting the standard errors with the dispersion parameter. We used a Laplace approximation to estimate model parameters. The effects of the fixed variables and their interactions were evaluated by comparing concurrent models (constructed by sequentially deleting the effect of interest) using likelihood ratio tests. This process was repeated until the minimal adequate model was obtained [37, 38]. Considering that plants (shrubs and trees) varied slightly in total height (mean = 1.98 ± 0.41 m) and that small herbaceous plants were not part of our sampling universe, we did not include plant size as a covariate. Models were constructed with the glmmADMB package [39]. To compare between categories of significant terms in the final model we performed contrast comparisons by using the *phia* package [40]. All analysis were performed in R environment V.3.02 [41].

To verify whether composition of the visiting ant fauna differs between EFN-bearing plants and EFN-lacking plants in both seasons, we used non-metric multidimensional scaling (NMDS), followed by an analysis of similarities (ANOSIM) [42]. Ant composition on plants was compared between plants with and without EFNs for each season by using the number of visited plants per transect according to each ant species recorded.

## Results

**Fig 1:**
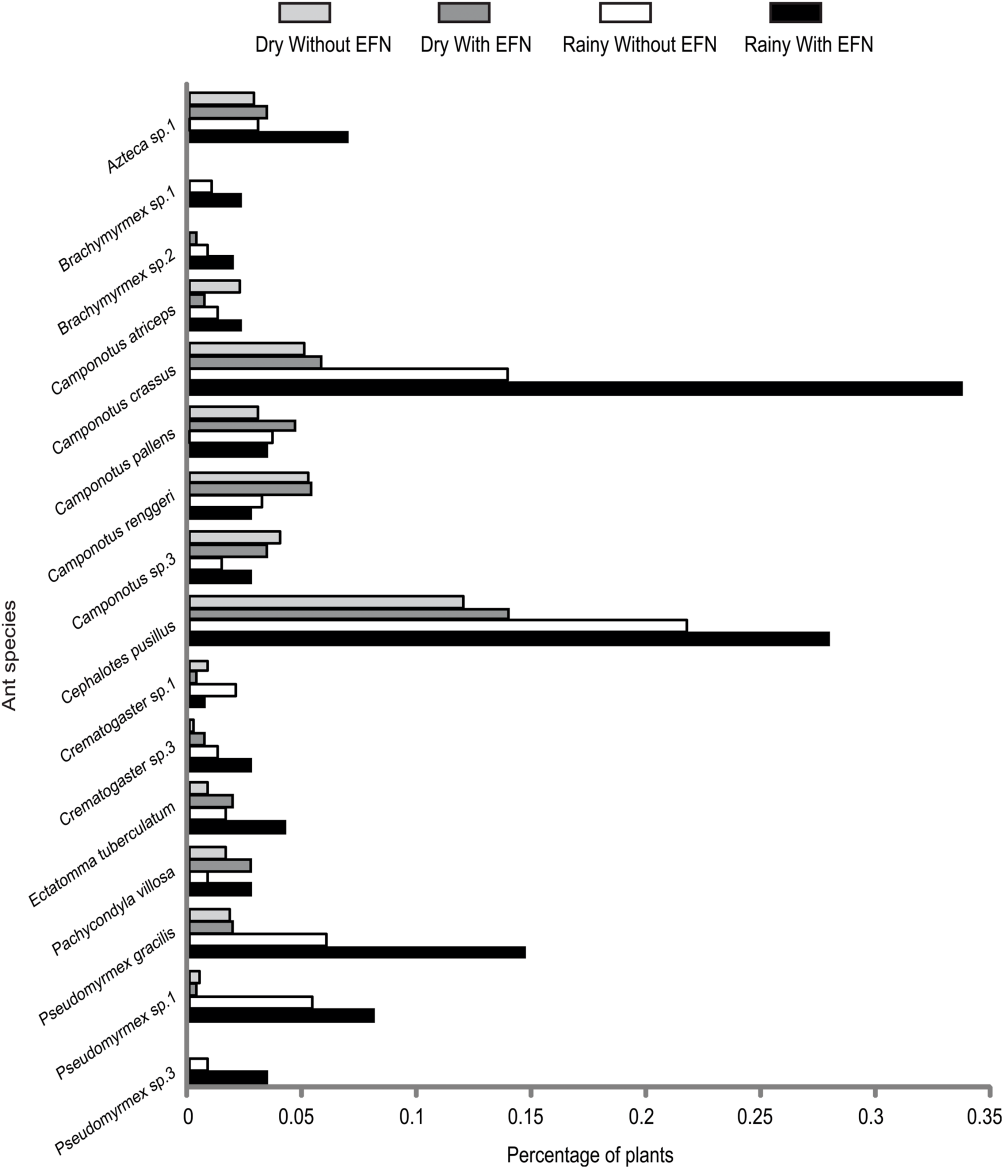
Percentage of Cerrado plants on which each ant species was found in each season. Plants were divided according with the presence (N = 266) or absence (N = 496) of EFNs for each season. Figure shows only ant species with ten occurrences or more.

Ant censuses revealed 38 species (36 in rainy and 26 in dry season, S1 Table) on 72 plant species (among these, 29% have EFNs = 21 species, S2 Table). In total, 4,955 ants were observed (78% in the rainy season = 3,868 individuals) on 762 plants (among these, almost 35% have EFNs = 266 individuals). Most ants were observed during daytime and only 6.84% of individuals (= 339) were recorded during the night. The subfamily Formicinae was the most representative, possessing 14 species, followed by Myrmicinae, with 11 species. Ant species that showed the highest occurrences (i.e., number of plant individuals on which ant species was found on) were *Camponotus crassus*, *Cephalotes pusillus* and *Pseudomyrmex gracilis*, respectively (Fig 1), but *Azteca* sp.1 was the species with the highest abundance of ant workers (S1 Table), and *Camponotus atriceps*, *C*. *pallens* cf. and *C*. *renggeri* were strictly nocturnal.

All studied plants during the rainy season had leaves, while during the dry season 2.4% (= 18 plants) were bare of leaves (Table 1). In rainy season, young leaves were observed in 51.2% (= 390), old leaves in 77.2% (= 588) and flowers in 11% (= 84) of all plants. In dry season, young leaves were observed in 9.1% (= 69), old leaves in 94.3% (= 719) and flowers in 3.9% (= 30) of all plants.

**Table 1:** Number of plants according to phenological state across seasons.

Plants possessing	Without EFN		With EFN		Total (%)	
	Rainy	Dry	Rainy	Dry	Rainy	Dry
B	0	1	0	17	0	18 (2.4%)
O	176	416	126	194	302 (39.6%)	610 (80.1%)
Y	83	19	64	5	147 (19.3%)	24 (3.1%)
O + Y	122	34	40	8	162 (21.2%)	42 (5.5%)
O + Y + FL	26	0	2	0	28 (3.7%)	0
O + Y + FR	16	1	9	1	25 (3.3%)	2 (0.3%)
Y + FL	13	0	0	0	13 (1.7%)	0
Y + FR	8	0	3	1	11 (1.4%)	1 (0.1%)
Y + FL + FR	3	0	0	0	3 (0.4%)	0
Y + O + FL + FR	1	0	0	0	1 (0.1%)	0
O + FL	27	19	7	10	34 (4.5%)	29 (3.8%)
O + FR	16	5	15	30	31 (4.1%)	35 (4.6%)
O + FL + FR	5	1	0	0	5 (0.7%)	1 (0.1%)
Total	496	496	266	266	762	762

B = bare of leaves; O = old leaves; Y = young leaves; FL = flowers + floral buds; FR = fruits

**Fig 2:**
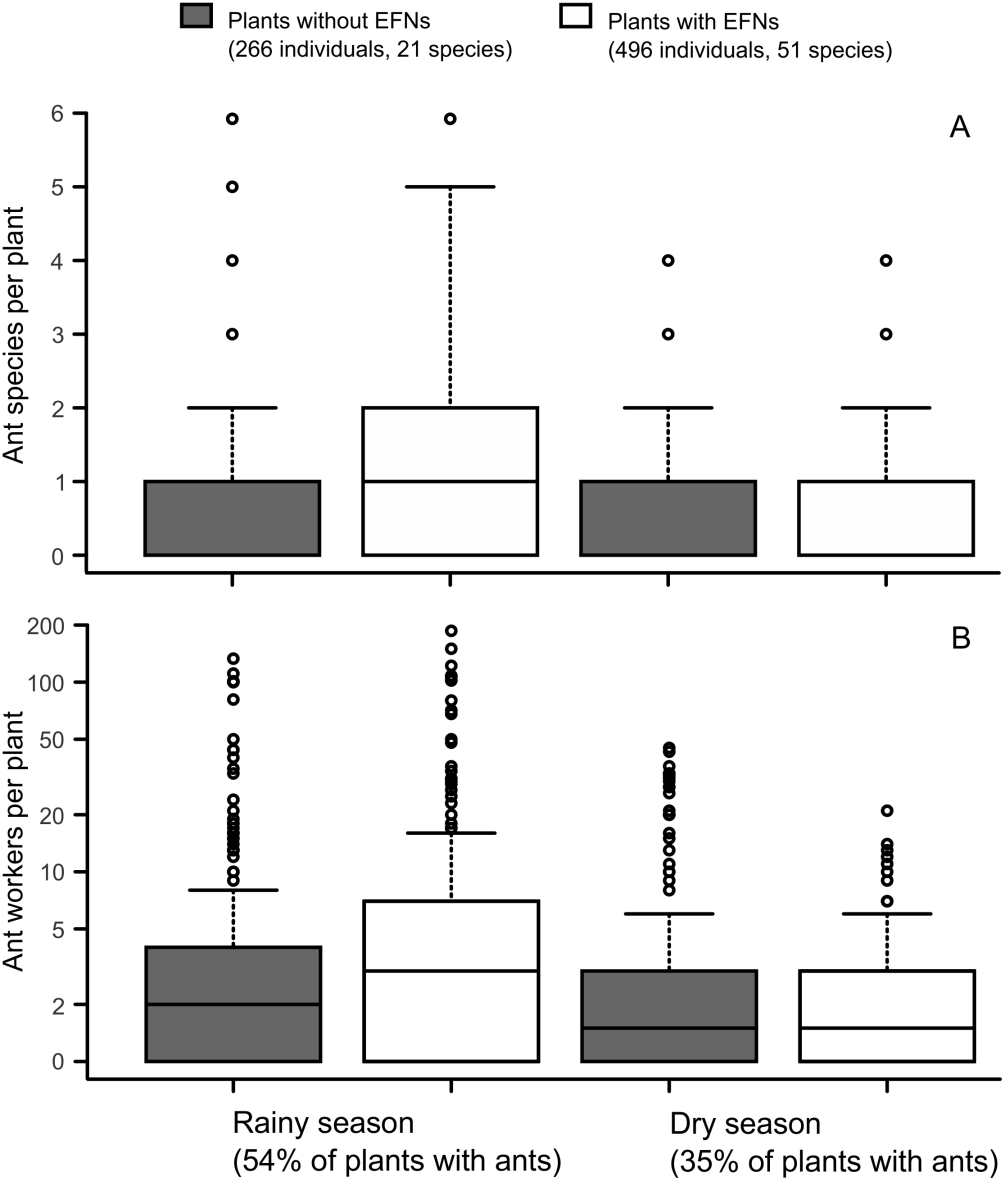
Seasonal ant visitation patterns on Cerrado plants with and without EFNs. Number of ant species (A) and ant workers (B) per plant. Black horizontal lines represent the median, boxes designate the second and third quartiles, and vertical bars indicate the range of data without outliers. Asterisks indicate significant differences (P < 0.5) between adjacent pairs of categories following contrast procedure.

**Fig 3:**
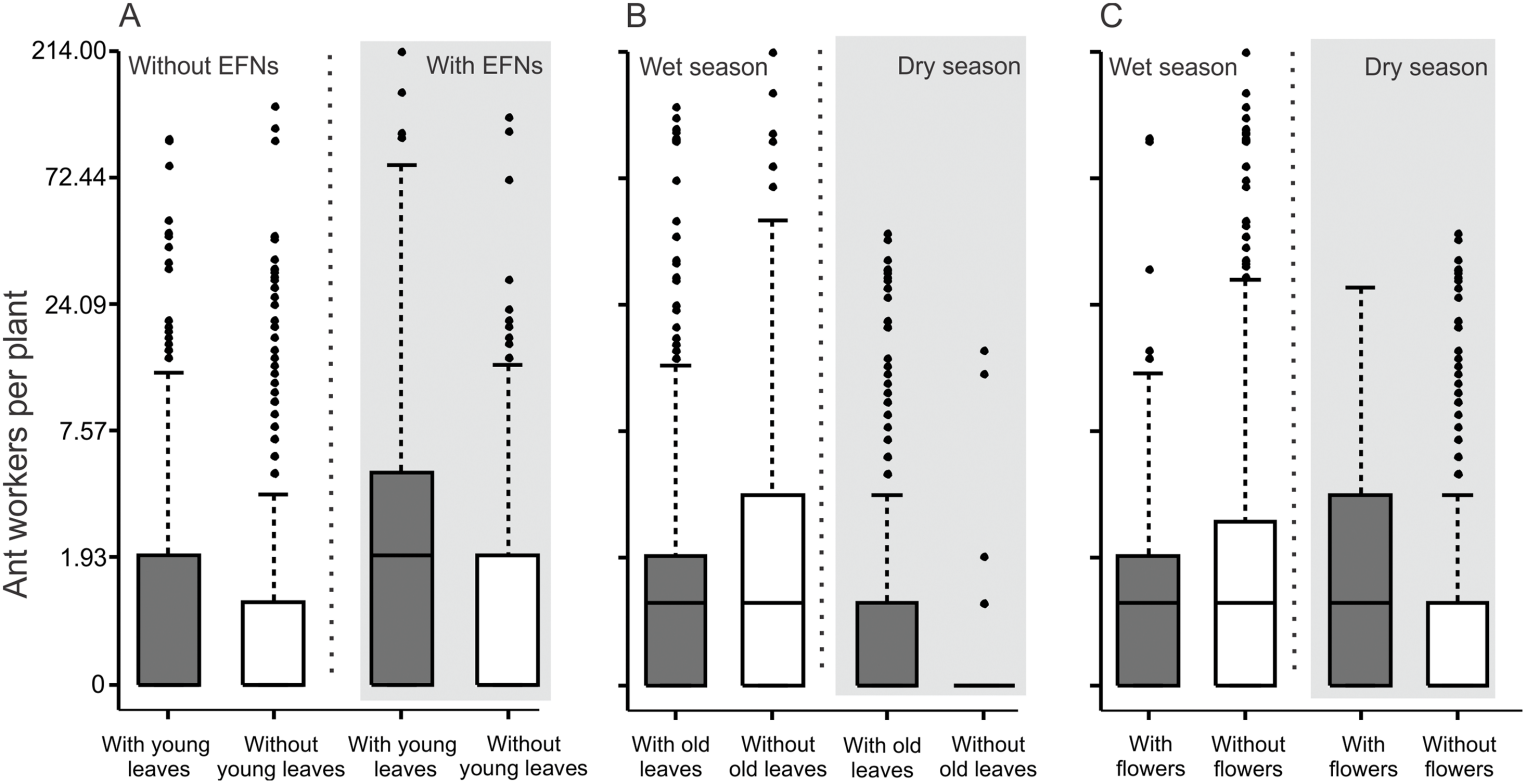
Ant visitation patterns on Cerrado plants according to phenological factors. Plants with and without old leaves according to the seasons (A), plants with and without flowers or floral buds according to the seasons (B). Black horizontal lines represent the median, boxes designate the second and third quartiles, and vertical bars indicate the range of data without outliers. Asterisks indicate significant differences (P < 0.5) between adjacent pairs of categories following contrast procedure.

**Fig 4:**
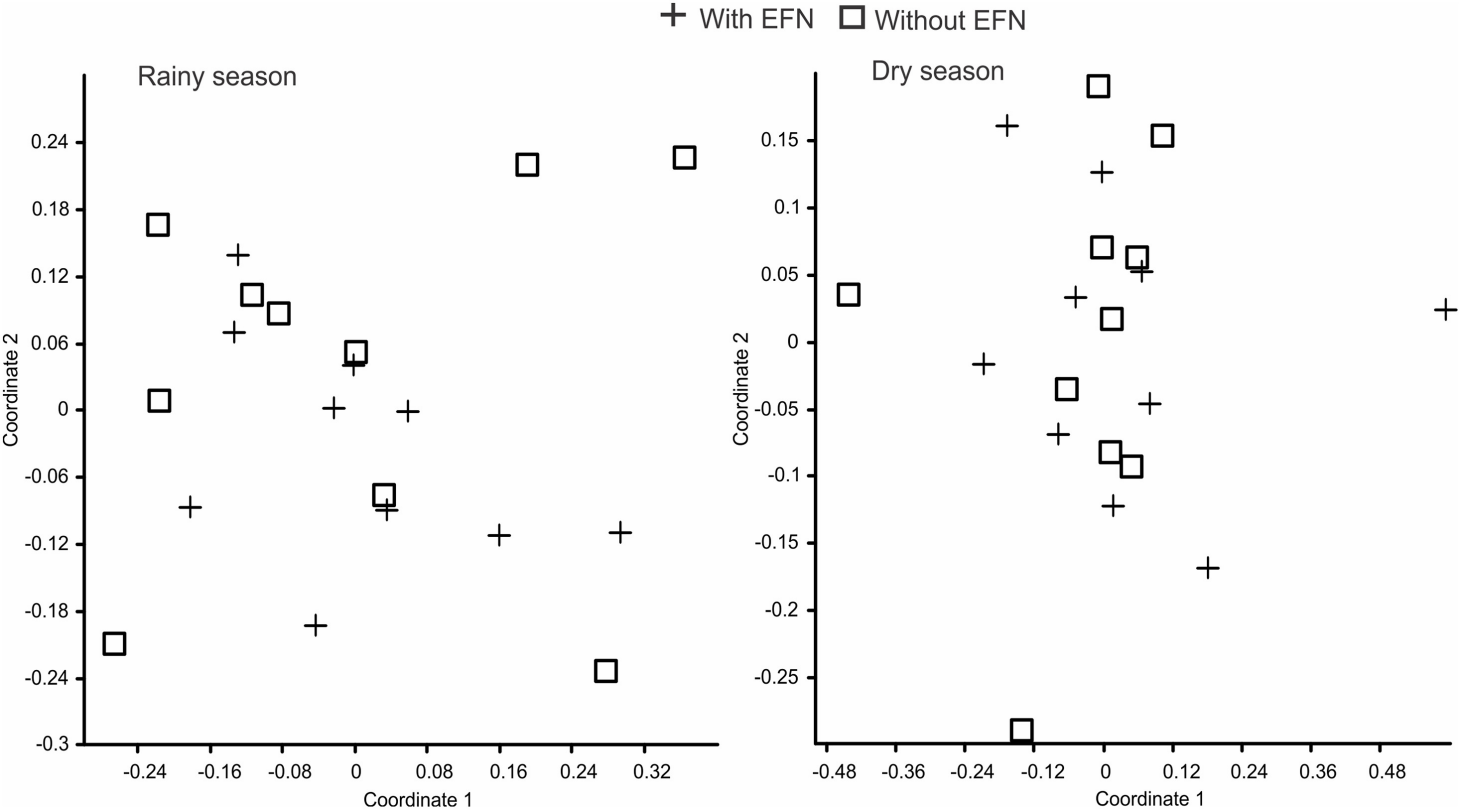
NMDS results obtained for the composition of ant species on Cerrado plants with and without EFNs (N = 10 transects) for both seasons. The composition of ant fauna did not differ (P > 0.05) between the groups according with ANOSIM tests.

Ant community was significantly affected by the interaction between seasonality and presence of EFNs: EFN-plants had higher ant richness (*X*^2^ = 28.34; *P* < 0.001; Fig 2A) and abundance (*X*^2^ = 5.35; *P* = 0.021; Fig 2B) during the rainy season, while in the dry period EFN presence did not influence ant visits (*X*^2^ < 0.8; *P* > 0.4; Fig 2; Table 2). Plant phenological factors were important in the models for ant richness and abundance. The presence of young leaves promoted ant richness (Table 2), and the presence of flowers and old leaves interacting with season were also important factors for increasing ant abundance on plants. More ants were observed on plants possessing old leaves (*X*^2^ = 10.49; *P* = 0.001; Fig 3A) during the dry period but not in the rainy period (*X*^2^ = 2.57; *P* = 0.11). A similar pattern was found for plants with flowers, which were more visited during the dry season (*X*^2^ = 3.84; *P* = 0.05; Fig 3B), but not in the rainy season (*X*^2^ < 0.01; *P* = 0.952). Composition of visiting ant fauna did not differ between plants with and without EFNs (Fig 4) in the rainy (Morisita index, NMDS stress = 0.19; ANOSIM *P* = 0.36) or dry season (Morisita index, NMDS stress = 0.17; ANOSIM *P* = 0.86).

**Table 2:** GLMM results showing the effect of EFNs and plant phenological factors on ant richness and abundance across seasons.

Random effect						
	SD			SD		
Transects	0.086			0.231		
Plant species	0.189			1.014		
Fixed effect	Richness			Abundance		
	Estimate ± SE	χ^2^	*P*-value	Estimate ± SE	χ^2^	*P*-value
Intercept	-0.441 ± 0.155	-	-	0.819 ± 0.309	-	-
Season (dry)	-0.428 ± 0.092	6.57	0.010*	0.472 ± 0.479	1.26	0.262
EFN (With)	0.496 ± 0.119	44.04	<0.001*	0.675 ± 0.330	6.36	0.011*
O (without)	-	0.14	0.704	0.620 ± 0.191	5.06	0.024*
Y (without)	-0172 ± 0.082	4.22	0.039*	-	2.66	0.103
FL (without)	-	0.80	0.371	-1.207 ± 0.410	0.13	0.721
FR (without)	-	0.00	0.951	-	0.12	0.729
Season (dry) x EFN (With)	-0.681 ± 0.164	13.30	<0.001*	-1.111 ± 0.235	22.05	<0.001*
Season (dry) x O (without)	-	0.85	0.355	-1.700 ± 0.456	14.26	<0.001*
Season (dry) x Y (without)	-	0.62	0.429	-	2.70	0.101
Season (dry) x FL (without)	-	3.53	0.060	-1.058 ± 0.496	5.44	0.012*
Season (dry) x FR (without)	-	0.13	0.970	-	0.02	0.887
Y (without) x EFN (With)	-	0.09	0.714	-	1.36	0.243
O (without) x Y (without)	-	0.09	0.756	-	2.86	0.091

The table shows the estimated coefficients for the linear model for each variable or category (standard-error) and the results of likelihood ratio test. The coefficient of intercept of the models represent estimates for rainy season, plants without EFNs and with all the phenological conditions.

N = 1,524 observations

SD = standard-deviation

SE = standard-error.

O = old leaves; Y = young leaves; FL = flowers + floral buds; FR = fruits

* Significant difference

There were few plants (< 30) possessing sap-sucking hemipterans (trophobiont insects) during the rainy season, however *Diospyros burchelli* (Ebenaceae), a species without EFNs, was considerably visited by ants due to aggregations of an unidentified Aleyrodidae (Sternorrhyncha) species found on all *D*. *burchelli* plants (= 17). In the dry season, fewer plants possessed the aggregations. Due to the scarcity of trophobionts, comparisons of ant-trophobiont interactions between plants with and without EFNs were not possible.

## Discussion

### Ant richness and abundance, EFN presence and seasonality

Presence and activity of EFNs, among other factors, would be decisive in the capacity of myrmecophilic plants to attract ants [5, 43, 44]. As extrafloral nectar is important for ant survival, growth and vitality of colonies [21, 45], we assumed that ant richness and abundance on plants are higher where and when the availability of this resource is greater. This was corroborated by our results that showed higher ant visitation on EFN-bearing plants compared to EFN-lacking plants in the rainy season, but not in the dry season, suggesting that the effects caused by EFNs on ant-plant interactions depend on season. This suggestion finds support in several studies that indicated temporal variations in ant-plant interactions [46–48], including ones in tropical savannas [32, 49].

Camarota and co-workers [34] also observed higher ant richness on plants with EFNs compared to those without, but there was no difference in ant richness per tree between periods of low and high EFN activity, for both EFN-bearing and EFN-lacking plants. Under circumstance of high hydric stress, water of extrafloral nectar (besides sugar) can be considered an important reward to ants (e.g. [50]), and the lack of this resource can affect ant survival during the dry season. This may maintain the richness of visiting ants during dry periods, but not necessarily a high number of foragers (abundance). Some ant colonies in acacia trees, for example, can die due to the lack of foliar nectar and Beltian bodies in the dry season [51].

Also, Schoereder and co-workers [8] studied another cerrado area and observed more ants in the dry season than in the rainy season; they suggested that EFNs would be more important for ants during the dry period. This assumption was reinforced by the hypothesis that, due to the low availability of soil water content, nectar produced in the dry season would be more concentrated than the nectar produced in the rainy period, and therefore sugars and amino acids would be in higher concentration in nectar, which would become more attractive to foraging ants [52].

The divergence between our results and those of Schoereder and co-workers [8] may have been caused by climate differences that affected the collection periods. Our data were collected within the first four months of each season (from October to January in the rainy period, and from April to July in the dry period) in an area that has climate classified as “Cwa”. Differently, that study was carried out in an area that has climate classified as “Cwb”, with rains concentrated between November-March and the dry period lasting up to seven months [53]. It is known that in the beginning of dry period ants can be still abundant on plants if lack of water and other environmental limitations associated with dry conditions are not yet strong. Also, it is possible to find young leaves sprouting out on plants in the end of dry season. As dates of field work were not reported by Schoereder and co-workers [8], the divergence of results can be understood if we presume that they considered dry season from August to October and rainy season from February to April. Besides difference in dry season timing between the studies, variations in the flora composition and plant phenological state (leading to different proportions of plants possessing young leaves and active EFNs) could have influenced results. In our study area *Q*. *multiflora*, *Ouratea hexasperma*, *O*. *spectabilis* and *Caryocar brasiliense* were the plant species with the highest occurrences, contrasting to *Q*. *grandiflora*, *Q*. *parviflora*, *Stryphnodendron adstringens* and *Lafoensia pacari*, which stood out in the registers of that study.

The identity of interacting species is relevant to explain the conditional outcomes in ant-plant systems [11, 54–56]. We observed that the genera *Camponotus*, *Cephalotes*, *Pseudomyrmex* and *Azteca* were represented among the most common ants on plants, especially on those possessing EFNs, as seen in other tropical regions and cerrado areas [5, 20, 34, 57–59]. Our results represent basically the foraging pattern of diurnal ant species, since these were far more sampled than the nocturnal ones. This is possibly due to differences between our sampling methods in day versus night, however, it must be highlighted that certain species are strictly diurnal (e.g,[60]). Therefore, additional investigation is necessary to confirm that the observed patterns are also valid for nocturnal ant species.

### Extrafloral nectar, plant phenology and seasonality

There are evidences that the outcomes of ant-plant-herbivore interactions are strongly influenced by plant phenology [13, 32]. In our study, plant phenology affected ant abundance and richness in different ways, and more plants with young leaves were observed in the rainy season compared to the dry season. Coinciding with this, Lange and co-workers [32] reported, in the same area, a higher percentage of plants with active EFNs during the rainy season. Indeed, our records showed not only that EFN-bearing plants were more visited but also that young leaves were promoters of ant visitation (number of species), indicating that plants in this circumstance may be more attractive to ants. It can be inferred that our study strengthens the “protection against herbivores hypothesis” (*sensu* [61]) to explain the role of EFNs as plant biotic defense. In the pre-reproductive phase, plants become more susceptible to herbivore visitation by showing young leaves [62, 63].

We observed that EFN presence only influences ant visitation on plants during the rainy season, but not during the dry season. Based on this, we can assume that, in the rainy season, when extrafloral nectar is abundant on vegetation, the consumption of this resource would be greater due to its greater availability over this period. On the contrary, when extrafloral nectar is produced in low quantity or quality (e.g. during the dry season), ants are obligated to search for other alternatives sources of food and water on vegetation [9, 26, 29]. This assumption is supported by our results, which showed plants with old leaves and flowers being more visited (possessing higher ant abundance) than those with young leaves or bare of leaves and flowers during the dry period.

Records of plants with young leaves being visited often by ants during the rainy season are common for many seasonal tropical ecosystems [5, 22, 27, 29, 32], and specifically for cerrado a peak of insect abundance in this period has already been observed for various insect orders [23]. Additionally, the beneficial effect of a higher ant presence on plants causing the reduction of herbivory and/or increase in the number of flowers/fruits has been demonstrated for several cerrado plant species (references in [6, 20, 64, 65]). These evidences suggest that the secretion of extrafloral nectar could be related to the existence of a peak in the abundance of herbivorous insects during the rainy period [20, 33], when vegetative growth of most plants occurs. Alternatively, the offer of extrafloral nectar could be related to the availability of soil water content, which would explain the higher volume of nectar secreted over the rainy period. However, Bixenmann and co-workers [62] suggest that, in the tropics, the production of extrafloral nectar is induced as a response to the ant presence and higher luminosity, but not by herbivores.

Considering that some plants become bare of leaves and few plants have young leaves during the dry period, it can be inferred that plants remaining with old leaves and possessing flowers or floral buds may increase in importance, because they represent sources of additional food items, such as prey herbivorous insects [66], floral tissues or nectar [67]. Although there are studies indicating that ants are infrequent visitors to flowers due to several reasons (references in [13, 68]), other studies recorded ant presence on flowers and other reproductive parts of plants in lowland dry tropics, semiarid areas, Mediterranean regions, alpine environments and Atlantic Rainforest (references in [5, 69]).

Although we did not investigate flower visitation by ants in this study, our results suggest that it is possible for floral tissue or nectar act as an attractive resource, especially in contexts of reduction/absence of extrafloral nectar. This suggestion is based on our finding that during the dry season ants tended to visit plants with flowers instead of those without flowers. Besides this, ants were seen several times feeding on flowers during the sampling.

Studies about the mechanisms of nectar production and regulation of its secretion are scarce [13] and necessary, especially for cerrado plants, to clarify the relation between extrafloral nectar availability, abundance/composition of arthropod fauna and seasonality. As nectar composition determines the spectrum of potential consumers, because animals differ in their nutritive preferences [13], investigations to compare quantity and quality of nectar in cerrado plants through seasons are also important to elucidate the effects on composition of arthropod fauna attracted to extrafloral nectar.

Although the present study was performed in only one year, it is reasonable to expect that the observed patterns may be similar across years, considering that the periodicity of leaf emergence and fall is well identified for cerrado plant species (see references in [70, 71]), and that the seasonality of cerrado savanna is strongly marked [72]. For instance, studies on herbivorous insects using *Caryocar brasiliense*, a common cerrado species, as a host plant showed that the leaf phenology registered in the first sampling was similar to the second one carried out approximately ten years later [63, 33].

### Composition of ant fauna on plants with and without EFNs

It is known that ants prefer nectar rich in amino acids [73], showing preferences for specific amino acids [52, 74, 75]. Considering the existence of valuable resources on plants with EFNs [21], we expected in our results that the composition of visiting ant fauna would be different between plants with and without EFNs, especially in periods when EFN is the main available food source for ants. This expectation was also based on the fact that preferences for sugars and amino acids between ants might vary according to their nutritive needs and kinds of mutualisms [75, 76]. However, previous studies had verified that ant species that visit EFN-bearing plants and EFN-lacking plants are, basically, the same [8, 34, 59]. Our results corroborate that ant species composition on plant community is not affected by EFN presence, even during rainy season when extrafloral nectar is more abundant on plants.

Several possibilities could explain the resemblance in the composition of ant fauna between plants with and without EFNs at cerrado vegetation. Firstly, connections between plants (bearing and lacking EFNs) favored by overlaps of crowns that bring together trunks and branches of different plants (which is very common at cerrado), would promote a shared visiting fauna. This explanation is supported by the study of Powell and co-workers [77], carried out in another cerrado area, where they demonstrated that plants with crowns more connected to others are richer in ant species than plants less connected. Secondly, composition of ant fauna on plants may be determined by the identity of plant species, size or overlapping instead of EFN presence [34]. However, it is possible to find ant nests more frequently in vegetation spots possessing only EFN-bearing plants than in spots possessing only EFN-lacking plants, but the frequency of which ant nests are found in vegetation spots possessing both kind of plants can be similar to those of spots having only plants with EFNs [78]. Additionally, there are evidences indicating niche differentiation for ant foraging behavior as a promoter of coexistence of ants in the same cerrado plants [77]. For instance, ant species vary in the time period that they use to visit a plant, therefore, several ant species may share the same space [60], and interspecific competition for resources such as EFNs may have a weak effect on ant community structure [34, 59]. Also, the availability of several resources on plants (e.g., trophobionts, insect prey or even flowers) would allow ant species to use both plants with or without EFNs [59, 79, 80].

We observed very few aggregations of trophobiont insects on plants during the rainy season (and even less in the dry season), thus, comparisons of ant-trophobiont interactions between plants with and without EFNs were not possible. This may suggest that trophobionts have low influence on the studied community. However, previous studies showed that the honeydew produced by trophobionts has a role in structuring the visiting ant community of cerrado plants [59, 81]. It can be inferred that in areas with high abundance of trophobiont insects and especially during dry season, when extrafloral nectar is produced in low quantity or quality, the presence of other liquid food sources (rather than EFNs) may determine the ant community structure.

## Conclusion

According to Díaz-Castelazo and co-workers [48], the facultative nature of the interactions between ants and plants, among other characteristics, associated to seasonal variation and disturbance, provides a richer community maintaining biodiversity. Our study, similarly to others (see examples in [5, 34]), indicated that ant-plant interactions mediated by extrafloral nectar are highly generalized. The low fidelity displayed by ants to resources of myrmecophilic plants, in other words, the facultative nature of these interactions, has been well documented [5, 12, 36, 82], and it can be inferred that “supergeneralist” species contribute to the total cohesion of interaction networks [58, 83]. In this study, we showed that plant visitation by ants is markedly influenced by seasonality and availability of extrafloral nectar.

We conclude that ant presence varies on cerrado vegetation through time as a consequence of changes in the availability of resources across seasons. However, these changes are not restricted to differential production of extrafloral nectar but also to variations in the phenological conditions of plants. Chamberlain and Holland [84] argued that context dependent effects are not common in ant-plant protection mutualisms, because ant effects on plants are consistently positive and rarely neutral or negative. Nevertheless, they recognize that the extent of the context dependency on the results of these interactions will be better understood along gradients of abiotic and biotic factors (e.g. [85]). In this sense, our study shows that ant-plant interactions mediated by extrafloral nectar are context dependent and conditional upon seasonal variation, corroborating the evidences of abiotic factors modulating the intensity of relations between partners [46, 62, 86, 87]. These factors are relevant to explain how diversity of ant-plant interactions is maintained at cerrado savanna (e.g.[13]).

## Supporting Information

S1 Table List of ant species observed on studied plants. ^1^ Total abund = Total abundance of ants per season; ^2^ (% on EFN) = how much of the total ant abundance was observed on EFN-bearing plants; ^3^ Mean ± sd (n obs) = mean number ± standard-deviation of ants per plant when ant species was observed (number of observations); ^4^ Period: (M) morning, (A) afternoon, (N) night.
(DOCX)

S2 Table List of studied plant species. ^1^ According to [8, 16]; ^2^ N indiv = number of plant individuals; ^3^ Ant abundance per plant = mean ± standard-deviation of the total number observed once in all periods (morning, afternoon and night) of each season.
(DOCX)
