# Supplementary material for: Temporal Variation in the Abundance and Richness of Foliage-Dwelling Ants Mediated by Extrafloral Nectar
Source: PLoS One. 2016 Jul 20;11(7):e0158283. doi: 10.1371/journal.pone.0158283 (PMC4954677; doi:10.1371/journal.pone.0158283)
Supplement: S1 Table — 1 Total abund = Total abundance of ants per season; 2 (% on EFN) = how much of the total ant abundance was observed on EFN-bearing plants; 3 Mean ± sd (n obs) = mean number ± standard-deviation of ants per plant when ant species was observed (number of observations); 4 Period: (M) morning, (A) afternoon, (N) night. (DOCX) [file pone.0158283.s001.docx]

S1 Table. List of ant species observed on studied plants.

| Subfamily | Rainy season | Dry season | Period^4^ |
| --- | --- | --- | --- |
| *Species* | Total Abund^1^ (% on EFN)^2^ | Total Abund^1^ (% on EFN)^2^ |  |
|  | *Mean ± sd (n obs)^3^* | *Mean ± sd (n obs)^3^* |  |
| Dolichoderinae |  |  |  |
| *Azteca* sp.1 | 1,623 (61%) | 210 (26%) | M, A |
|  | *33.8 ± 39.7 (48 obs)* | *8.4 ± 10.1 (25 obs)* |  |
| *Azteca* sp.2 | 25 (12%) | 34 (0%) | M, A |
|  | *3.6 ± 2.8 (7 obs)* | *11.3 ± 16.2 (3 obs)* |  |
| *Dolichoderus laminatus* cf. Emery, 1894 | 1 (0%) | - | N |
|  | *(1 obs)* |  |  |
| *Dolichoderus lutosus* (Smith, 1858) | 2 (50%) | - | N |
|  | *1.0 ± 0.0 (2 obs)* |  |  |
| *Dorymyrmex* sp. | 2 (100%) | - | A |
|  | *(1 obs)* |  |  |
| Ectatomminae |  |  |  |
| *Ectatomma tuberculatum* (Olivier, 1792) | 23 (56%) | 13 (69%) | M, A, N |
|  | *1.0 ± 0.2 (22 obs)* | *1.4 ± 0.9 (9 obs)* |  |
| Formicinae |  |  |  |
| *Brachymyrmex* sp.1 | 49 (47%) | - | M, A |
|  | *4.1 ± 5.2 (12 obs)* |  |  |
| *Brachymyrmex* sp.2 | 143 (82%) | 1 (100%) | M, A |
|  | *15.9 ± 32.1 (9 obs)* | *(1 obs)* |  |
| *Camponotus atriceps* (Smith, 1858) | 16 (37%) | 29 (38%) | N |
|  | *1.3 ± 0.9 (12 obs)* | *2.2 ± 2.1 (13 obs)* |  |
| *Camponotus crassus* Mayr, 1862 | 407 (54%) | 100 (26%) | M, A |
|  | *2.1 ± 2.1 (193 obs)* | *2.4 ± 3.4 (42 obs)* |  |
| *Camponotus lespesii* Forel, 1886 | 5 (80%) | 7 (14%) | N |
|  | *1.0 ± 0.0 (5 obs)* | *1.4 ± 0.9 (5 obs)* |  |
| *Camponotus pallens* cf. (Le Guillou, 1842) | 35 (37%) | 37 (46%) | N |
|  | *1.3 ± 0.8 (27 obs)* | *1.4 ± 0.7 (27 obs)* |  |
| *Camponotus renggeri* Emery, 1894 | 27 (26%) | 69 (52%) | N |
|  | *1.2 ± 0.5 (23 obs)* | *1.7 ± 2.2 (40 obs)* |  |
| *Camponotus* sp.1 | 9 (22%) | - | M |
|  | *1.5 ± 0.8 (6 obs)* |  |  |
| *Camponotus* sp.2 | 3 (100%) | 1 (0%) | M, A |
|  | *1.5 ± 0.7 (2 obs)* | *(1 obs)* |  |
| *Camponotus* sp.3 | 16 (50%) | 39 (28%) | N |
|  | *1.1 ± 0.2 (15 obs)* | *1.3 ± 0.8 (29 obs)* |  |
| *Camponotus* sp.4 | 16 (62%) | 19 (37%) | N |
|  | *1.4 ± 1.2 (11 obs)* | *1.5 ± 1.2 (13 obs)* |  |
| *Camponotus* sp.5 | 3 (100%) | - | A |
|  | *(1 obs)* |  |  |
| *Camponotus* sp.6 | 3 (100%) | - | A |
|  | *(1 obs)* |  |  |
| *Camponotus* sp.7 | 1 (100%) | - | N |
|  | *(1 obs)* |  |  |
| Myrmicinae |  |  |  |
| *Cephalotes bruchi* (Forel, 1912) | 3 (100%) | 1 (100%) | M, A |
|  | *(1 obs)* | *(1 obs)* |  |
| *Cephalotes pusillus* (Klug, 1824) | 645 (56%) | 269 (27%) | M, A |
|  | *2.9 ± 4.1 (225 obs)* | *2.4 ± 3.4 (269 obs)* |  |
| *Crematogaster brasiliensis* Mayr, 1878 | 1 (0%) | 45 (0%) | M, A |
|  | *(1 obs)* | *22.5 ± 3.5 (2 obs)* |  |
| *Crematogaster* sp.1 | 282 (7%) | 83 (14%) | M, A |
|  | *18.8 ± 18.1 (15 obs)* | *11.8 ± 14.5 (7 obs)* |  |
| *Crematogaster* sp.2 | 85 (100%) | - | M, A |
|  | *21.2 ± 21.7 (4 obs)* |  |  |
| *Crematogaster* sp.3 | 120 (46%) | 32 (22%) | M, A |
|  | *8.0 ± 11.6 (15 obs)* | *8.0 ± 8.1 (4 obs)* |  |
| *Nesomyrmex spininodis* (Mayr, 1887) | 27 (0%) | 21 (0%) | M, A |
|  | *3.9 ± 5.0 (7 obs)* | *10.5 ± 13.4 (2 obs)* |  |
| *Ochetomyrmex semipolitus* Mayr, 1878 | - | 25 (0%) | A |
|  |  | *12.5 ± 3.5 (2 obs)* |  |
| *Pheidole oxyops* cf. Forel, 1908 | 17 (18%) | 5 (0%) | M, A, N |
|  | *2.1 ± 1.6 (8 obs)* | *2.5 ± 2.1 (2 obs)* |  |
| *Solenopsis* sp.1 | 65 (100%) | - | M, A |
|  | *32.5 ± 24.8 (2 obs)* |  |  |
| *Solenopsis* sp.2 | 22 (91%) | - | M, A |
|  | *7.3 ± 11.0 (3 obs)* |  |  |
| Ponerinae |  |  |  |
| *Pachycondyla villosa* (Fabricius, 1804) | 12 (67%) | 20 (40%) | M, A, N |
|  | *1.1 ± 0.3 (11 obs)* | *1.2 ± 1.0 (16 obs)* |  |
| *Pachycondyla* sp. | - | 4 (25%) | N |
|  |  | *1.0 ± 0.0 (4 obs)* |  |
| Pseudomyrmecinae |  |  |  |
| *Pseudomyrmex gracilis* (Fabricius, 1804) | 79 (53%) | 15 (47%) | M, A |
|  | *1.1 ± 0.4 (72 obs)* | *1.1 ± 0.3 (14 obs)* |  |
| *Pseudomyrmex unicolor* (Smith, 1855) | 2 (0%) | 1 (100%) | M, A |
|  | *1.0 ± 0.0 (2 obs)* | *(1 obs)* |  |
| *Pseudomyrmex* sp.1 | 61 (51%) | 4 (25%) | M, A |
|  | *1.2 ± 1.3 (49 obs)* | *1.0 ± 0.0 (4 obs)* |  |
| *Pseudomyrmex* sp.2 | 4 (25%) | 3 (67%) | M, A |
|  | *1.3 ± 0.6 (3 obs)* | *1.0 ± 0.0 (3 obs)* |  |
| *Pseudomyrmex* sp.3 | 34 (82%) | - | M, A |
|  | *2.3 ± 1.9 (15 obs)* |  |  |
| **Total** | **3,868 (55%)** | **1,087 (26%)** |  |
|  | **36 species** | **26 species** |  |
