# Supplementary material for: Temporal Variation in the Abundance and Richness of Foliage-Dwelling Ants Mediated by Extrafloral Nectar
Source: PLoS One. 2016 Jul 20;11(7):e0158283. doi: 10.1371/journal.pone.0158283 (PMC4954677; doi:10.1371/journal.pone.0158283)
Supplement: S2 Table — 1 According to [8, 16]; 2 N indiv = number of plant individuals; 3 Ant abundance per plant = mean ± standard-deviation of the total number observed once in all periods (morning, afternoon and night) of each season. (DOCX) [file pone.0158283.s002.docx]

**S2 Table. List of studied plant species.**

| Family | EFN Location^1^ | N indiv^2^ | Ant abundance per plant (mean ± sd)^3^ | |
| --- | --- | --- | --- | --- |
| *Species* |  |  | Rainy | Dry |
| Annonaceae |  |  |  |  |
| *Annona crassiflora* Mart. | - | 2 | 0.2 ± 0.4 | 0 |
| *Xylopia aromatica* (Lam.) Mart. | - | 4 | 4.1 ± 10.7 | 3.9 ± 11.7 |
| Apocynaceae |  |  |  |  |
| *Aspidosperma tomentosum* Mart. | - | 1 | 0 | 9.3 ± 13.6 |
| *Hancornia speciosa* Gomes | - | 2 | 0.5 ± 0.5 | 0 |
| Asteraceae |  |  |  |  |
| *Piptocarpha rotundifolia* (Less.) Baker | - | 7 | 0.3 ± 0.5 | 0.1 ± 0.3 |
| Bignoniaceae |  |  |  |  |
| *Handroanthus ochraceus* (Cham.) Mattos | Leaf blade | 1 | 0.7 ± 1.1 | 0 |
| Calophyllaceae |  |  |  |  |
| *Kielmeyera coriacea* Mart. & Zucc. | - | 1 | 16.7 ± 28.9 | 11.0 ± 16.5 |
| *Kielmeyera rubriflora* Cambess. | - | 1 | 0 | 0 |
| Caryocaraceae |  |  |  |  |
| *Caryocar brasiliense* Cambess. | Buds, shoot tips | 23 | 5.3 ± 14.8 | 0.4 ± 1.4 |
| Celastraceae |  |  |  |  |
| *Plenckia populnea* Reissek | - | 3 | 0.3 ± 0.7 | 0 |
| Chrysobalanaceae |  |  |  |  |
| *Couepia grandiflora* (Mart. & Zucc.) Benth. | - | 7 | 6.8 ± 21.7 | 1.6 ± 2.8 |
| *Licania humilis* Cham. & Schltdl. | Leaf blade | 13 | 1.2 ± 3.0 | 0.6 ± 1.9 |
| Connaraceae |  |  |  |  |
| *Connarus suberosus* Planch. | - | 15 | 0.1 ± 0.4 | 0.2 ± 0.6 |
| Dilleniaceae |  |  |  |  |
| *Davilla elliptica* A.St.-Hil. | - | 8 | 0.1 ± 0.3 | 0.1 ± 0.3 |
| Ebenaceae |  |  |  |  |
| *Diospyros burchellii* Hiern | - | 17 | 2.4 ± 4.8 | 1.5 ± 3.8 |
| Erythroxylaceae |  |  |  |  |
| *Erythroxylum deciduum* A.St.-Hil. | - | 23 | 0.2 ± 0.6 | 0.1 ± 0.3 |
| *Erythroxylum tortuosum* Mart. | - | 7 | 0.3 ± 0.7 | 0.3 ± 0.9 |
| Fabaceae |  |  |  |  |
| *Andira paniculata* Benth. | - | 1 | 0 | 0.3 ± 0.6 |
| *Bauhinia rufa* (Bong.) Steud. | Intra stipules, petiole | 10 | 0.6 ± 0.9 | 0.3 ± 1.3 |
| *Bowdichia virgilioides* Kunth | - | 3 | 0.8 ± 2.3 | 0.3 ± 0.7 |
| *Copaifera langsdorffii* Desf. | Leaf blade | 8 | 0.3 ± 0.9 | 0.2 ± 1.0 |
| *Dalbergia miscolobium* Benth. | - | 21 | 0.3 ± 0.8 | 0.1 ± 0.2 |
| *Hymenaea stignocarpa* Mart. ex Hayne | Leaf blade | 6 | 1.0 ± 1.9 | 0.4 ± 0.8 |
| *Leptolobium dasycarpum* Vogel | - | 6 | 0.2 ± 0.4 | 0.2 ± 0.4 |
| *Machaerium opacum* Vogel | - | 4 | 0.3 ± 0.8 | 0.1 ± 0.3 |
| *Plathymenia reticulata* Benth. | Stem | 21 | 4.3 ± 18.1 | 0.0 ± 0.2 |
| *Pterodon pubescens* (Benth.) Benth. | Rachis | 16 | 10.6 ± 29.3 | 0.7 ± 2.3 |
| *Stryphnodendron adstringens* (Mart.) Coville | Rachis | 8 | 0.2 ± 0.4 | 0.2 ± 0.5 |
| *Stryphnodendron polyphyllum* Mart. | Rachis | 13 | 1.1 ± 4.8 | 0.1 ± 0.4 |
| *Tachigali aurea* Tul. | - | 1 | 0 | 0 |
| *Vatairea macrocarpa* (Benth.) Ducke | - | 1 | 0.3 ± 0.6 | 0 |
| Lythraceae |  |  |  |  |
| *Lafoensia pacari* A.St.-Hil. | Leaf blade | 1 | 0.3 ± 0.6 | 0 |
| Malpighiaceae |  |  |  |  |
| *Banisteriopsis laevifolia* (A.Juss.) B.Gates | Leaf blade | 14 | 0.3 ± 0.8 | 0.3 ± 0.9 |
| *Banisteriopsis malifolia* (Nees & Mart.) B.Gates | Leaf blade | 6 | 0.6 ± 1.6 | 0.9 ± 3.3 |
| *Byrsonima basiloba* A.Juss. | - | 41 | 1.7 ± 9.5 | 0.4 ± 1.6 |
| *Byrsonima coccolobifolia* Kunth | - | 7 | 0.2 ± 0.4 | 0.1 ± 0.3 |
| *Byrsonima intermedia* A.Juss. | - | 30 | 0.3 ± 0.7 | 0.4 ± 1.3 |
| *Heteropterys pteropetala* A.Juss. | Leaf blade | 5 | 1.9 ± 4.6 | 0 |
| Malvaceae |  |  |  |  |
| *Eriotheca gracilipes* (K.Schum.) A.Robyns | Leaf blade, petiole | 15 | 1.9 ± 3.8 | 1.0 ± 1.9 |
| Melastomataceae |  |  |  |  |
| *Miconia ferruginata* DC. | - | 1 | 0 | 1.3 ± 1.1 |
| *Miconia leucocarpa* DC. | - | 3 | 0 | 0 |
| *Miconia ligustroides* (DC.) Naudin | - | 1 | 0 | 0 |
| *Miconia rubiginosa* (Bonpl.) DC. | - | 5 | 7.5 ± 26.0 | 0.1 ± 0.2 |
| Meliaceae |  |  |  |  |
| *Cabralea canjerana* (Vell.) Mart. | - | 11 | 1.2 ± 2.2 | 0.3 ± 0.8 |
| Moraceae |  |  |  |  |
| *Brosimum gaudichaudii* Trécul | - | 3 | 0.9 ± 2.0 | 2.2 ± 6.7 |
| Myrtaceae |  |  |  |  |
| *Blepharocalyx salicifolius* (Kunth) O.Berg | - | 27 | 0.6 ± 2.5 | 0.0 ± 0.1 |
| *Eugenia aurata* O.Berg | - | 1 | 0 | 0.7 ± 1.1 |
| *Eugenia calycina* Cambess. | - | 4 | 0.1 ± 0.3 | 0.2 ± 0.6 |
| *Eugenia punicifolia* (Kunth) DC. | - | 33 | 0.3 ± 0.8 | 0.0 ± 0.2 |
| *Myrcia splendens* (Sw.) DC. | - | 6 | 7.5 ± 24.8 | 0.4 ± 0.9 |
| *Myrcia variabilis* DC. | - | 8 | 0.2 ± 0.6 | 0 |
| *Myrcia* sp. | - | 1 | 5.0 ± 8.7 | 0 |
| *Psidium* spp. | - | 2 | 0 | 0 |
| Nyctaginaceae |  |  |  |  |
| *Guapira graciliflora* (Mart. ex Schimidt) Lundell | - | 29 | 2.2 ± 7.2 | 0.7 ± 3.9 |
| *Guapira noxia* (Netto) Lundell | - | 8 | 4.9 ± 20.3 | 2.7 ± 7.3 |
| *Neea theifera* Oerst. | - | 21 | 0.1 ± 0.4 | 0.6 ± 3.9 |
| Ochnaceae |  |  |  |  |
| *Ouratea hexasperma* (A. St.-Hil.) Baill. | Cataphylls (stipules) | 27 | 2.3 ± 6.1 | 0.2 ± 0.7 |
| *Ouratea spectabilis* (Mart.) Engl. | Cataphylls (stipules) | 26 | 0.6 ± 2.4 | 0.1 ± 0.4 |
| Primulaceae |  |  |  |  |
| *Myrsine guianensis* (Aubl.) Kuntze | - | 4 | 8.5 ± 20.0 | 5.4 ± 9.9 |
| Proteaceae |  |  |  |  |
| *Roupala montana* (Aubl.) | - | 30 | 0.3 ± 1.4 | 0.3 ± 1.6 |
| Rubiaceae |  |  |  |  |
| *Tocoyena formosa* (Cham. & Schltdl.) K.Schum. | Leaf blade, calyx | 2 | 1.3 ± 1.0 | 0 |
| Rutaceae |  |  |  |  |
| *Hortia brasiliana* Vand. ex DC. | - | 2 | 2.0 ± 2.2 | 1.7 ± 2.6 |
| Salicaceae |  |  |  |  |
| *Casearia sylvestris* Sw. | - | 8 | 0.4 ± 0.9 | 0.1 ± 0.3 |
| Sapindaceae |  |  |  |  |
| *Matayba guianensis* Aubl. | - | 3 | 0.2 ± 0.4 | 0.3 ± 0.5 |
| Sapotaceae |  |  |  |  |
| *Pouteria ramiflora* (Mart.) Radlk. | - | 14 | 0.7 ± 1.8 | 1.2 ± 3.4 |
| *Pouteria torta* (Mart.) Radlk. | - | 13 | 0.7 ± 1.9 | 0.8 ± 3.3 |
| Styracaceae |  |  |  |  |
| *Styrax ferrugineus* Nees & Mart. | - | 36 | 1.0 ± 3.1 | 0.4 ± 2.0 |
| Symplocaceae |  |  |  |  |
| *Symplocos rhamnifolia* A.DC. | - | 1 | 5.3 ± 8.4 | 0 |
| Vochysiaceae |  |  |  |  |
| *Qualea grandiflora* Mart. | Stem, petiole, peduncle | 7 | 1.9 ± 3.9 | 0.2 ± 0.7 |
| *Qualea multiflora* Mart. | Stem, petiole, peduncle | 38 | 3.5 ± 11.8 | 0.5 ± 1.4 |
| *Qualea parviflora* Mart. | Stem, petiole, peduncle | 6 | 2.0 ± 3.3 | 0.1 ± 0.3 |
| *Vochysia cinnamomea* Pohl | - | 8 | 0.2 ± 0.6 | 0.2 ± 0.4 |
| Total |  | 762 |  |  |
